# Simultaneous time-varying viscosity, elasticity, and mass measurements of single adherent cancer cells across cell cycle

**DOI:** 10.1038/s41598-020-69638-z

**Published:** 2020-07-30

**Authors:** Olaoluwa O. Adeniba, Elise A. Corbin, Anurup Ganguli, Yongdeok Kim, Rashid Bashir

**Affiliations:** 1 Department of Mechanical Science and Engineering, University of Illinois Urbana-Champaign, Urbana, IL 61801 USA; 2 Micro and Nanotechnology Laboratory, University of Illinois Urbana-Champaign, Urbana, IL 61801 USA; 3 Biomedical Engineering Department, University of Delaware, Newark, DE 19716 USA; 4 Materials Science and Engineering Department, University of Delaware, Newark, DE 19716 USA; 5 Nemours/Alfred I. duPont Hospital for Children, Wilmington, DE 19803 USA; 6 Department of Bioengineering, University of Illinois Urbana-Champaign, Urbana, IL 61801 USA; 7 Department of Materials Science and Engineering, University of Illinois Urbana-Champaign, Urbana, IL 61801 USA; 8 Carle Illinois College of Medicine, University of Illinois Urbana-Champaign, Urbana, IL 61801 USA

**Keywords:** Biophysical methods, Lab-on-a-chip, Cancer

## Abstract

Biophysical studies on single cells have linked cell mechanics to physiology, functionality and disease. Evaluation of mass and viscoelasticity versus cell cycle can provide further insights into cell cycle progression and the uncontrolled proliferation of cancer. Using our pedestal microelectromechanical systems resonant sensors, we have developed a non-contact interferometric measurement technique that simultaneously tracks the dynamic changes in the viscoelastic moduli and mass of adherent colon (HT-29) and breast cancer (MCF-7) cells from the interphase through mitosis and then to the cytokinesis stages of their growth cycle. We show that by combining three optomechanical parameters in an optical path length equation and a two-degree-of-freedom model, we can simultaneously extract the viscoelasticity and mass as a function of the nano-scaled membrane fluctuation of each adherent cell. Our measurements are able to discern between soft and stiff cells across the cell cycle and demonstrated sharp viscoelastic changes due to cortical stiffening around mitosis. Cell rounding before division can be detected by measurement of mechanical coupling between the cells and the sensors. Our measurement device and method can provide for new insights into the mechanics of single adherent cells versus time.

## Introduction

Cellular viscoelasticity is directly associated with physiological and pathological states of the cell. Mechanical phenotypes are signatures of cellular functions and behaviors that are related to human health and diseases. It is commonly known that cancer cells are softer than their normal counterpart^1,2^. The stiffness of cancer cells has been associated with pronounced invasiveness due to their increased deformability^3,4^, which, when coupled with reduced adhesion^5,6^, enables cancer cells to easily move through other tissues and metastasize. Viscoelasticity of single cells affects how they interact with their microenvironment, and the discrepancies in their physical properties may influence mechano-signaling pathways and in turn, affect cellular behavior^7,8^ and growth^9,10^. Recent work in the cardiovascular sciences has shown the direct effects of viscoelasticity and force output using drug-induced systems^11,12^. Changes in the mechanical properties of cell, in particular cell elasticity, has been associated with cytoskeletal organization. The cytoskeleton is an interconnected filamentous network that is responsible for organizing cellular content, interfacing with the external environment, and coordinating forces for movement and shape. Recent work has shown that both internal and external forces act through the cytoskeleton to influence the mechanical properties and behavior of cells as they progress through the growth cycle (interphase and mitosis)^13,14^. It is well known that mammalian cells become round and ‘ball up’ during mitosis, abandoning their elongated morphology, and then after mitosis they return to their original shape^15^. The cytoskeletal process associated with mitotic cell rounding has been thoroughly investigated, however, the link between cytoskeleton and the changes in mechanical properties over the cell cycle is under explored.

Changes in cell cycle mechanics can aid in better understanding of disease, however, the heterogeneity of cell cycle dynamics within a population proves to be challenging, as this requires the ability to handle, manipulate, and analyze individual cells. The rise of many techniques to study mechanical properties (e.g., viscoelasticity) of cells has led to an increase in understanding of mechanical properties and disease, and include methods such as atomic force microscopy (AFM)^2,16,17^, micropipette aspiration^13,18,19^, and magnetic twisting cytometry. These techniques probe the behavior of cells at different length and time scales, and employ different stress–strain magnitudes and behaviors. However, other biomarkers like cell mass is believed to be an important physiologic parameter that if dysregulated could give rise to disease^20^. Considering cell mass is determined not only by the cytoskeleton but by the contents within such as water and proteins, tracking changes in cell mass and viscoelasticity may provide key insights for understanding the changes in cellular structure, response, and function. Other techniques have emerged to measure mass change over the cell cycle, such as quartz crystal microbalance (QCM)^21^, suspended microchannel resonator (SMR)^3,22^, picobalance^23^, quantitative phase imaging (QPI)^20^. Most of these are limited by the inability to estimate time-varying elasticity and viscosity moduli alongside mass measurements of a whole cell over time^24^.

Our work presents, the first investigation of a time-varying simultaneous estimation of viscoelasticity and mass for individual adherent cells over the cell cycle. We achieve this by adding a temporal dimension to our recently developed micro-resonator based technique for measuring the biophysical properties of individual adherent cells. Using this technique, we are able to quantify the nanoscale membrane fluctuations (height oscillations) of individual live human colon cancer cells (HT-29) and human low metastatic breast cancer cells (MCF-7) over the cell cycle. We are able to observe the mitotic rounding event in the viscoelastic properties. The ability of our technique to resolve viscoelastic properties and mass simultaneously can help to elucidate intricate dynamics of the cell cycle.

## Results

### Non-destructive mapping of cell viscoelasticity

**Figure 1:**
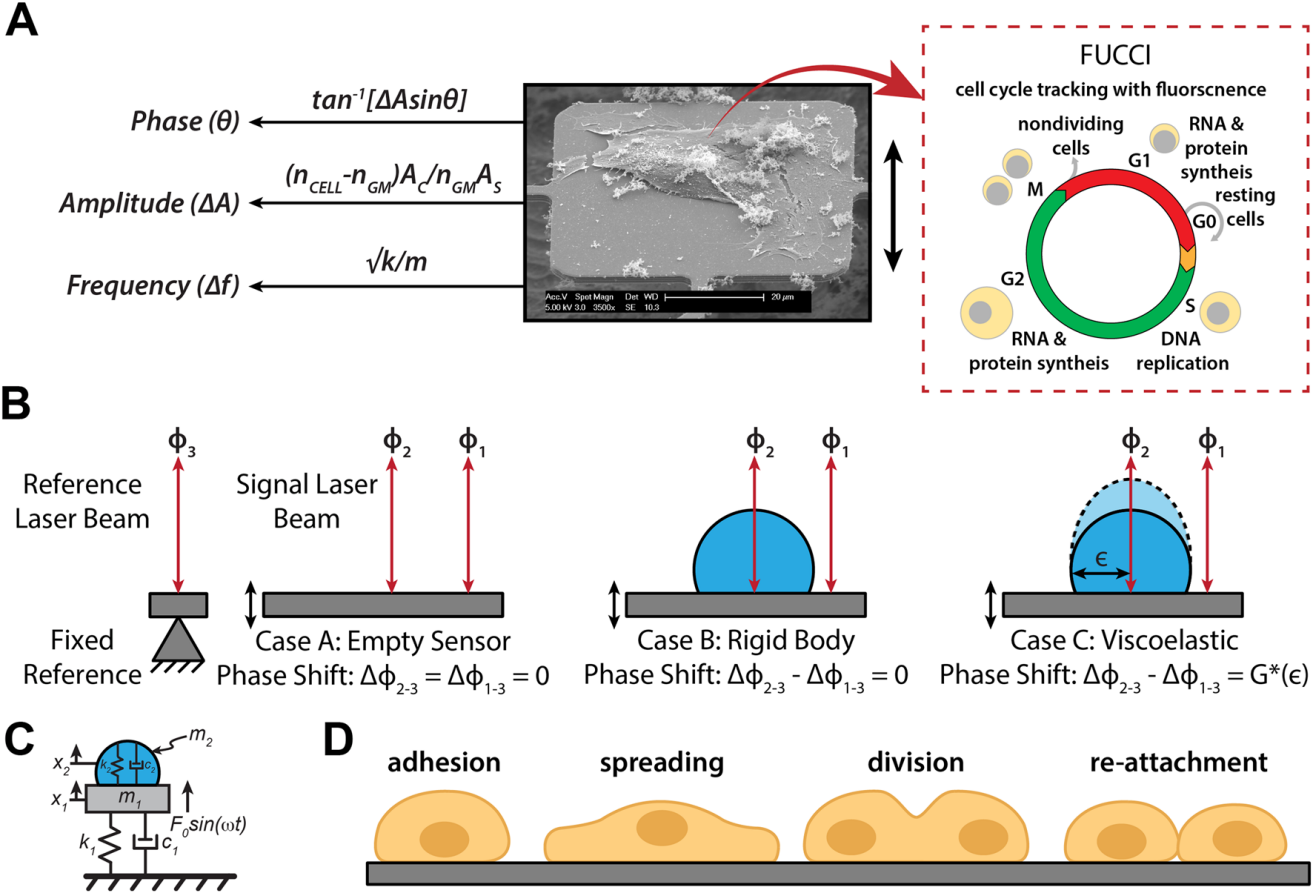
Overview of measurement scheme. (**A**) Summary of the vibration induced phase shift ($\Delta \phi ),$amplitude ratio, $\left( {\Delta A} \right),$ and frequency shift $(\Delta f$) relate to the viscoelastic properties and mass of the cell. These parameters are extracted during the vibration of a cell-loaded sensor while the cell cycle stage progression is being observed through phases G_1_–S–G_2_–M. (**B**) Cross-sectional figures elucidating the vibration induced phase shift, $\Delta \phi$ of a targeting signal beam, $\phi_{1}$ and $\phi_{2}$ on an empty sensor; inside and outside a rigid cell; and inside and outside of a viscoelastic cell; compared to a reference beam, $\phi_{3}$. G*(є) represents the cell dynamic moduli (viscoelastic moduli) as a function of lateral position, є, which tracks the cell heights oscillation. (**C**) Model of sensor-cell system as a 2-DOF suspended mass model where the cell mass (*m*_*2*_) is considered a Kelvin-Voigt viscoelastic solid with elastic stiffness (*k*_*2*_) and viscous coefficient (*c*_*2*_) connected to the sensor, and the sensor mass (*m*_*1*_) is connected to the fixed substrate by a second Kelvin-Voigt spring-damper (*k*_*1*_*, c*_*1*_). The model assumes an oscillatory force F(t) applied to the sensor mass. (**D**) Cell physiological transition from initial adhesion through cell rounding/division to reattachment to a patterned surface.

In this work, we can repeatedly measure the viscoelastic moduli and mass changes corresponding to cell growth, proliferation, and mitotic shape changes of single HT-29 and MCF-7 cells. We used a MEMS resonant sensor consisting of a 60 × 60  µm^2^ platform suspended by four beam springs that are arrayed in a 9 × 9 format of 81 sensors (Fig. 1A). The sensors operate with the aid of electromagnetic stimulation generating Lorentz force actuation and producing an out-of-plane motion in the first resonance mode. Similar to previous studies^1,24–27^, the velocity of the sensor vibration is monitored and measured by a laser Doppler vibrometer (LDV) in conjunction with a lock-in amplifier to capture our sensor responses (see Supporting Information). Our system observables—the frequency (${\varvec{f}})$, amplitude $\left( {\varvec{A}} \right)$, and optical phase shift $\left( {\Delta \phi } \right)$**—**are collected from the LDV measurements to extract the mass and viscoelastic values for individual cells versus time. The measurements were carried out in three schemes. First, the responses of an unloaded vibrating sensor (in air) was measured to determine the spring constant. Second, we measured the responses of an unloaded sensor (in media) to determine a reference resonant frequency and amplitude for subsequent comparative analysis. Third, the sensor was loaded with a live cell, and frequency and amplitude shifts are collected over time. During live cell measurements, the laser is alternately passed through the cell and outside the cell to measure the variations in the optical path length (OPL). The laser path length changes due to height oscillations of the cell during vibration and results in a phase shift (Fig. 1B–C)^1,24–27^. The observed change in the OPL correlate directly to the material and structural properties of the cell. These measurements are performed over time to capture the growth progression of each cell from an initial adherence stage through spreading, division (retraction), and reattachment (Fig. 1D). The viscoelastic effect of HT-29 cells on the resonant frequency and amplitude of the sensor were previously quantified to generate a large space of potential viscoelastic moduli solutions^24^. By incorporating the OPL shift as an additional variable, we can examine the morphological and phenotypical heterogeneity of each cell on a cell-by-cell basis.

### Mechanical viscoelastic properties change as cells grow

**Figure 2:**
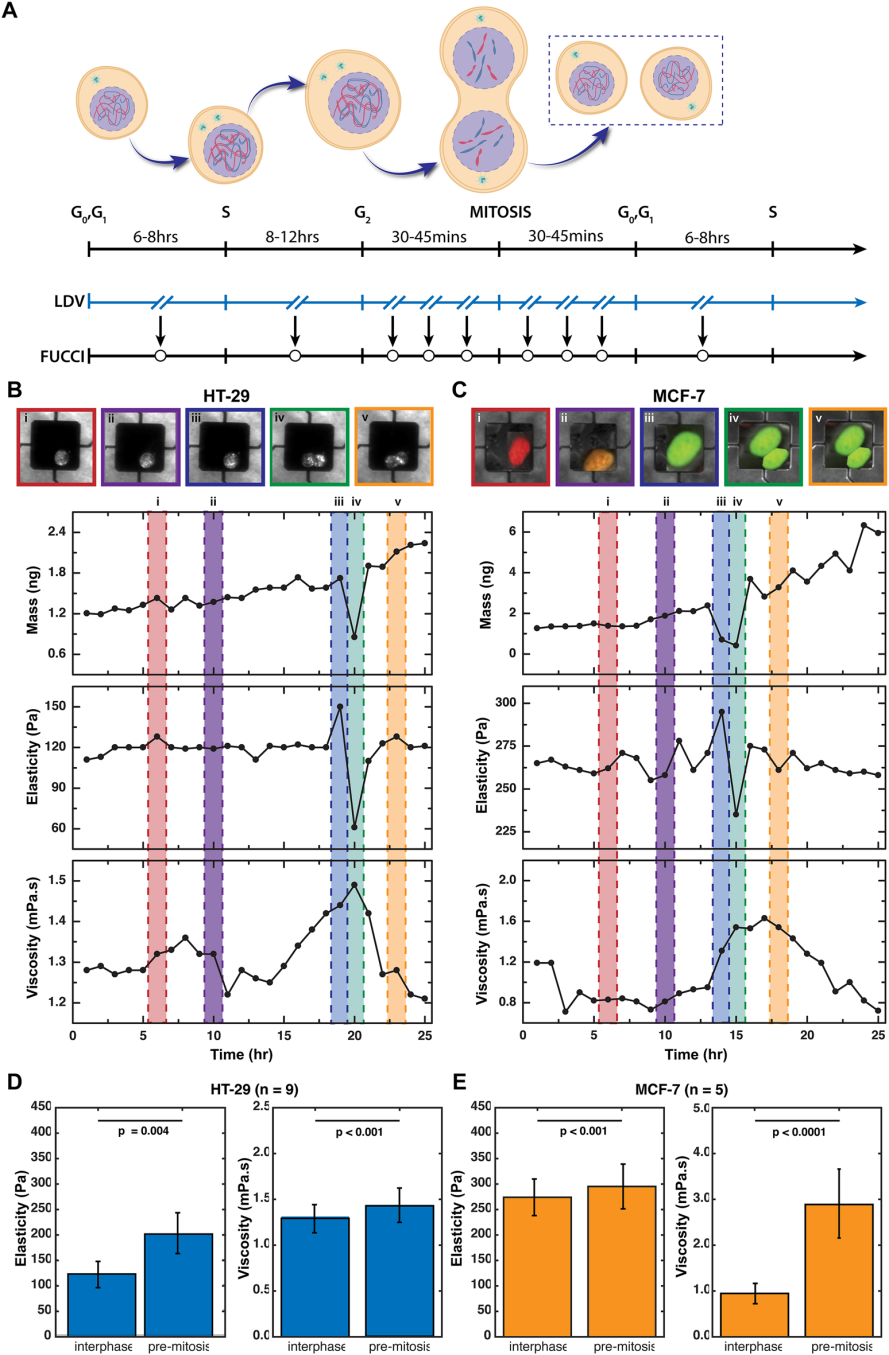
Mechanical viscoelastic properties change as cells grow. (**A**) Experimental timeline highlighting the transition of cell optomechanical measurement on sensors with respect to the LDV position while carrying out fluorescence imaging. (**B**–**C**) Cell mass, elasticity, and viscosity versus time for both (**B**) HT-29 and (**C**) MCF-7. For single cell growth analysis of HT-29 and MCF-7 cells, the mass and stiffness data was analyzed for the interphase (i, ii), prior to mitosis (iii) and after a mitotic event (iv and v). Division is shown with the individual cells and daughter cells. These parameters are extracted during the vibration of a cell-loaded sensor while the cell cycle stage progression is being observed through phases G_1_ (Red)–S (Orange)–G_2_ (Green)–M (Green) using FUCCI cell cycle reporter. (**D**–**E**) Comparison of average interphase elasticity and viscosity values for both (**D**) HT-29 (n = 9) and (**E**) MCF-7 (n = 5) cells during interphase (average) versus pre-mitosis (iii). Data presented as a mean ± standard deviation.

**Figure 3:**
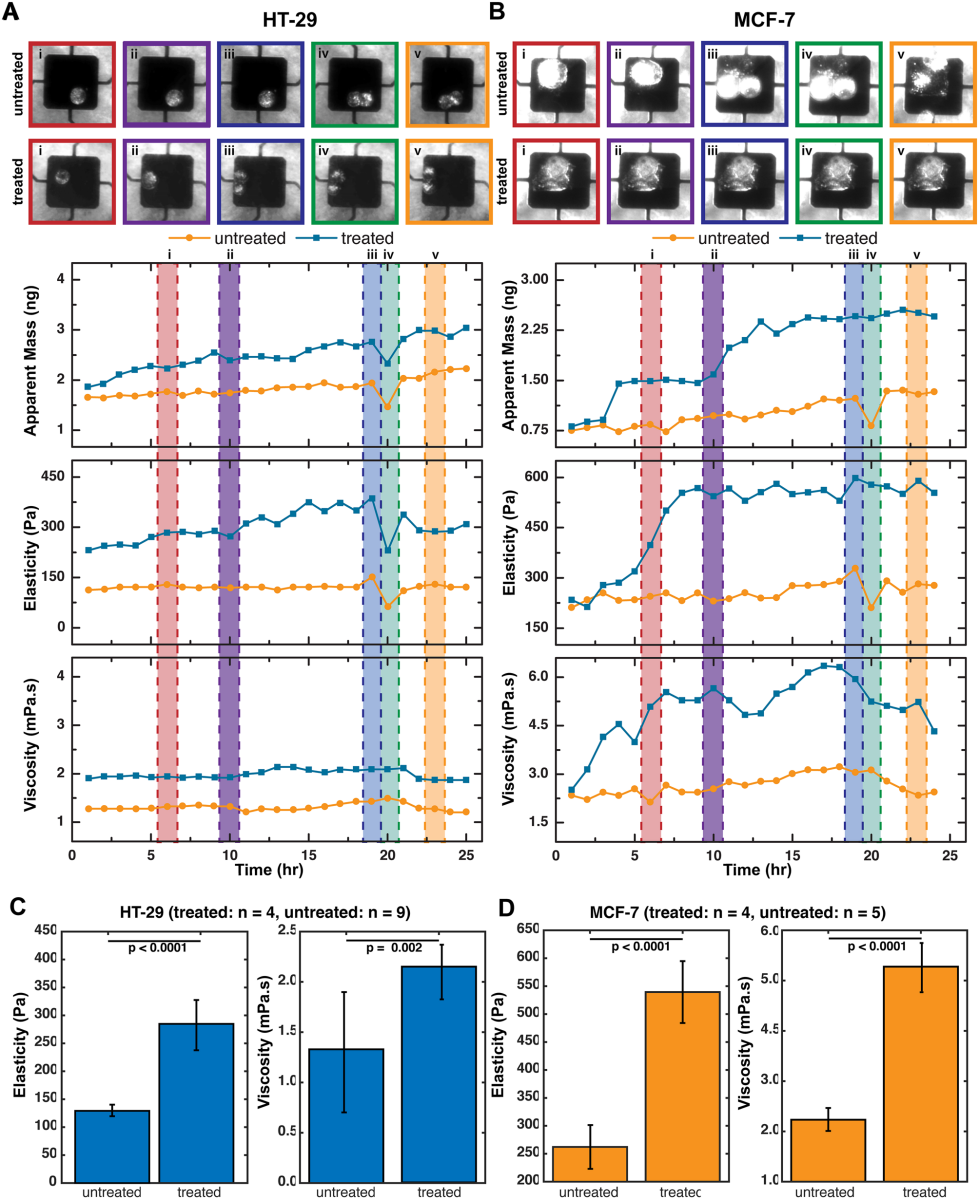
RhoA significantly increases viscoelasticity. (**A**) Time-Varying Mass, Elasticity and Viscosity measurement of RhoActivator—mediated HT-29 cells (treated) and unmodified HT-29 cells (untreated) measured by our MEMS resonator. Plot show a significant increase in cell viscoelastic moduli of modified HT-29 cells due to phosphorylation of actin. (**B**) Comparison between plot showing a significant increase in cell viscoelastic moduli of modified MCF-7 cells due to phosphorylation of actin fibres. Dashed lines are used to trace the mass and viscoelastic values per cell cycle stage. MCF-7 remains in the G_2_ checkpoint phase before apoptosis. (**C**–**D**) Comparison of average Elasticity and Viscosity values for RhoActivator—mediated (treated) and untreated (**D**) HT-29 (treated: n = 4, untreated: n = 9) and (**E**) MCF-7 (treated: n = 4, untreated: n = 5) cells during interphase. Plot shows a significant difference in viscoelastic moduli for treated cells versus untreated cells. Data presented as a mean ± standard deviation.

Investigating temporal events across the cell cycle requires single cell analysis within the larger population. The cell cycle is a highly regulated process but also dependent on environmental parameters and morphological changes (Fig. 2A). Prior to cell division (Fig. 2B–C, markers i/ii), we found the baseline viscoelastic values of ~ 152 ± 30 Pa and ~ 1.1 ± 0.3 mPa s for HT-29 cells as well as ~ 260 ± 80 Pa and ~ 1.4 ± 0.8 mPa s for MCF-7 cells (summarized in Tables 1 and 2). These findings agree with previous reports of average viscoelastic measurements^1,28–30^. At the onset of mitosis (marker iii), a cell undergoes changes due to its architecture (morphology), the cell attachment area is decreased resulting in the shape becoming round, which is necessary for subsequent mitotic events (i.e. spindle morphogenesis)^31^. This process of cell rounding involves rearrangement of the actin cytoskeleton, reduction of adhesion, and an increase in cortical rigidity (Fig. 1D). Considering the dynamics around cell division (mitosis, marker iii, untreated), we observe a decrease in stiffness at the mitotic entry, which also suggests a decrease in inertial coupling between the cells and the substrate (Figs. 2 and 3A). However, upon entry into mitosis, the cortical actin stiffens, and the cell becomes round to facilitate division, thereby causing a reduced cell height oscillation during sensor vibration. The occurrence of this peak elasticity value suggests that the cell cortical stiffening effects outweighs any potential effect that reduction in cell attachment could possibly cause before mitosis.

**Table 1:** Summary of elasticity on Fig. 4 for HT-29 and MCF-7 cells in comparison to average values from previous studies.

Average elasticity values (Pa) from previous studies	HT-29 (~ 152 ± 60.0)	MCF-7 (~ 260 ± 80.0)
i	120 ± 40.5	270 ± 80.1
ii	129 ± 41.4	280 ± 85.6
iii	150 ± 59.1	315 ± 49.0
iv*	40 ± 15.2	210 ± 18.4
v	119 ± 35.0	240 ± 25.1

*indicates mass dip.

**Table 2:** Summary of viscosity on Fig. 4 for HT-29 and MCF-7 cells in comparison to average values from previous studies.

Average viscosity (mPa s) from previous studies	HT-29 (~ 1.1 ± .3)	MCF-7 (~ 1.4 ± .8)
i	1.3 ± 0.2	1.2 ± 0.4
ii	1.33 ± 0.9	0.9 ± 0.4
iii	1.44 ± 0.7	1.6 ± 0.5
iv*	1.50 ± 0.8	1.61 ± 0.4
v	1.21 ± 0.39	0.9 ± 0.5

*indicates mass dip.

The increase in cortical rigidity prior to mitosis, (pre-mitosis) is directly observed (Fig. 2D–E) stage iii—across both cell types as a significant (1.1–1.6 fold) increase in the viscoelastic values. Previous studies^31–33^ have shown similar increases in cortical rigidity and associated moesin^21^ (a protein activated upon entry into mitosis), as well as cell rounding when entering mitosis. Furthermore, to highlight this pronounced viscoelastic phenomena, Fig. 2D–E presents a paired t-test to compare the viscoelasticity values during pre-mitosis against an average of the entire interphase spectrum. The results reveal a statistically significant (*p* ≈ 0.001) difference in elasticity between interphase values: (129 ± 41 Pa for HT-29; 266 ± 78 Pa for MCF-7) and pre-mitosis values (213 ± 73 Pa for HT-29; 297 ± 94 Pa for MCF-7). Likewise, a comparison in viscosity shows a high statistically significant differences (*p* < 0.001) between interphase values: (1.31 ± 0.32 mPa s for HT-29; 0.92 ± 0.17 mPa s for MCF-7) and pre-mitosis values (1.44 ± 0.42 mPa s for HT-29; 2.89 ± 0.79 mPa s for MCF-7). At the point of division during mitosis, a sharp decrease in apparent cell mass is observed in our studies (marker iv). This decrease in apparent mass during mitosis is caused by the cell temporarily decreasing its contact area^24,26^, thus leading to a reduction of the inertial loading of the cell and causing a higher strain rate and viscosity. Subsequently, there is a corresponding apparent decrease in cell elasticity, a function of the cell’s reduced inertial coupling. After exiting mitosis, the baseline viscoelastic values are restored to the pre-mitosis values (marker v). Prior studies suggest that there exists tensional homeostasis through contractions to counterbalance forces along the stress fibers that help cell attachment and re-attachment to surfaces after mitosis, potentially regulating this return to baseline viscoelasticity^14,34^.

### Drug-induced increase of actin polymerization increases viscoelastic properties as cells grow

**Figure 4:**
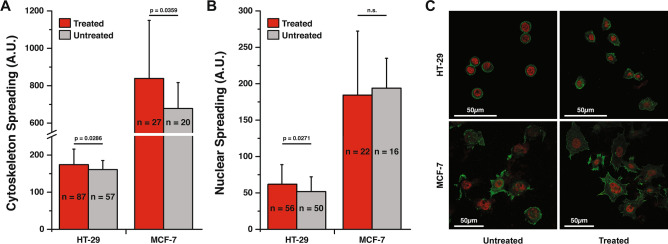
HT-29 (colon) and MCF-7 (breast) cancer cell spreading response to Rho Activator. Quantification for HT-29 and MCF-7 treated and untreated cells with the Rho Activator for their: (**A**) Cytoskeleton Spreading (**B**) Nuclear Spreading. Plot shows cell area (spreading) of both cell lines increased with the addition of the Rho activator, which is indicative of increased actin polymerization. Data presented as a mean ± standard deviation. (**C**) Representative images of HT-29 and MCF-7 treated and untreated with the Rho Activator comparing cytoskeletal and nuclear spread.

Rho GTPases are key regulators necessary to maintain the dynamics of cell shape during cell cycle progression and also at the onset of mitosis. To further investigate the structural changes within the cell and the influence on viscoelasticity, we quantified changes in mass and viscoelastic properties of both cell types over the cell cycle when the cytoskeletal structural components were specifically manipulated using a Rho activator. Treatment with a Rho activator, a direct agent that stimulates actin polymerization, increased the viscoelastic moduli as compared to the untreated samples over the time course (Fig. 3). This agrees well with established work involving of Rho addition to cells^33,35^. Stimulating actin polymerization through Rho-mediation causes an increase the stiffness, an increase in the inertial coupling of the cells with the sensor, and reduction in growth rate of the cell. For validation, we imaged representative cells with and without Rho activator and found that there was a significant increase (using a t-test) in cell area (spreading) of both cell lines with the addition of the Rho activator (Fig. 4), which is indicative of increased actin polymerization.

When the HT-29 and MCF-7 cell lines are treated with a Rho activator, we observe an increase in all mechanical measures. If we average the baseline changes between Rho-treated and untreated for mass, elasticity, and viscous properties, we see ~ 2.5-, ~ 3-, and ~ 3-fold increases, respectively. We expect an increase in the stress fiber development with Rho activation^33^^,^therefore, we infer that since there is more tension created by the stress fibers and a higher focal adhesion by the increased cell area, resulting in a reduced membrane fluctuation (structural deformation) and a higher apparent mass reading due to an increased cell-sensor coupling. To further characterize our mass measurement sensitivity and ensure reproducibility, we have also carried out 100 repeated mass measurements on rho-treated and untreated HT-29 and MCF-7 cells as shown in the Supplementary information Fig. S6. Consistent noise levels of HT-29 cells (untreated: 2.46 ± 0.16 ng, treated: 4.67 ± 0.32 ng) with MCF-7 cells (untreated: 3.41 ± 0.11 ng, treated: 3.78 ± 0.11 ng) show that our mass measurements are minimally sensitive to these rho-mediated cell-sensor noise levels for both cell lines fall within (~ 10%) one order of magnitude of their signal measurement.

Figure 3E–F presents a t-test comparison of viscoelasticity values between rho-treated and untreated cells. Statistically significant (*p* < 0.0001) differences were observed in the elasticity of untreated cells: (128 ± 14 Pa for HT-29; 247 ± 36 Pa for MCF-7) against treated cells: (281 ± 43 Pa for HT-29; 248 ± 57 Pa for MCF-7). Likewise, a comparison of viscosity values shows significant differences (*p* < 0.0001) between untreated cells (1.21 ± 0.60 mPa s for HT-29; 2.67 ± 0.36 mPa s for MCF-7) and treated cells (2.14 ± 0.32 mPa s for HT-29; 5.28 ± 0.41 mPa s for MCF-7). These results are consistent with prior studies^33,35^ which have revealed that compared to their non-treated counterparts, Rho-activator-mediated cancer cells are stiffer, and that this cell stiffness is dependent on actin polymerization and the activation of stress fibers, potentially increasing the spring and damper-like behaviors of the constituent actin fibers. These studies showed through fluorescent staining that the cells spread differently and wider due to the activation of these stress fibers^33^. Interestingly, as shown in Fig. 2F we observed that the MCF-7 Rho-mediated cell does not show any signs of division possibly due to the fact that Rho is introduced in the G_1_ or S checkpoint phase; the cell gets stuck there by affecting division and activating apoptosis after a prolonged arrest^36^. Conversely, Fig. 2E and Supplementary information Fig. S3–S4 show evidence of division with rho-treated MCF-7 and HT-29 cells, indicating that cell cycling might not be affected depending on the growth phase the Rho-drug is introduced.

### Local stiffness variations within a cell over mitosis

**Figure 5:**
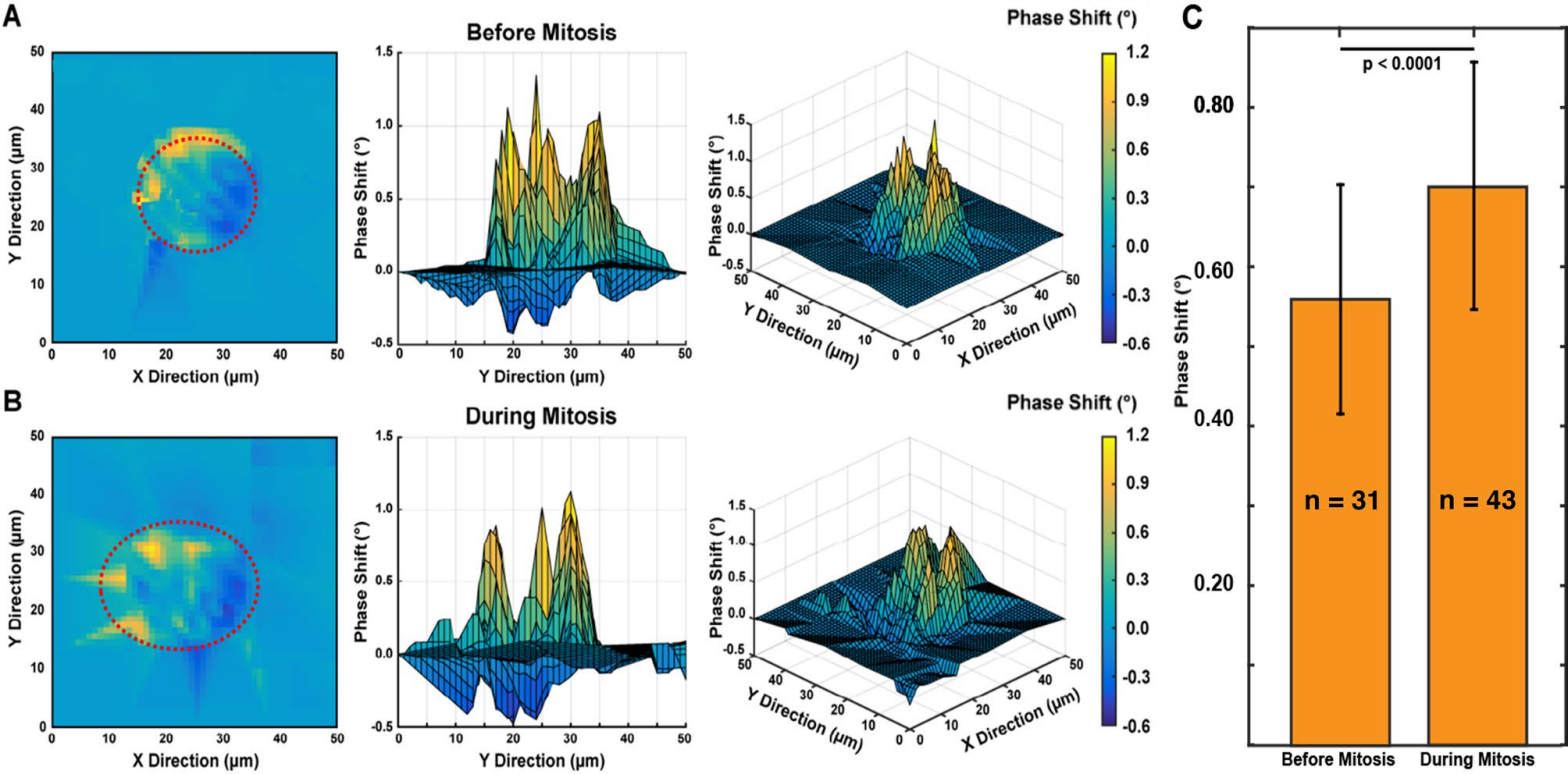
Scanning measurement maps of vibration induced phase shifts (VIPS) of an HT-29 cell. These maps indicate stiffness differences of a HT-29 cell at different stages of the cell cycle. Top, side, and 3D views of the same live cell both (**A**) prior to and (**B**) during mitosis. Prior to mitosis, we observed an increase in stiffness and an average inside cell (dotted red lines) lower phase shift (VIPS) of 0.56 ± 0.21°. During mitosis, cells are partially detached—cell height oscillation increases (with softness); hence a higher inside cell (dotted red lines) VIPS of 0.75 ± 0.31° is observed. (**C**) Bar chart showing statistically significant differences (*p* < 0.0001) in the pre-mitosis (n = 31) and during-mitosis (n = 43) phase shift (VIPS) values. Data presented as a mean ± standard deviation.

We are interested in measuring changes in local mechanical properties across the cell cycle, which may exhibit different temporal patterns than the bulk properties, especially around mitosis. To investigate the short-term viscoelastic response spatially across the cell, our resonant sensors are used as a vibrating substrate to initiate a vibration induced phase shift (VIPS)^27^. While the platform of our mass sensor is oscillating at a fixed frequency, the phase of the substrate’s velocity is measured using the LDV and the lock-in-amplifier. A cell vertically vibrating on a substrate experiences a structural deformation due to oscillation of its height, and the degree of the height oscillation is inversely proportional to the cell stiffness. We scanned the entire cell and sensor, and observed a negligible VIPS value for areas outside the cell, whereas a phase increase is observed inside the cell, though with several peaks in phase across the cell area. In Fig. 5A–B, the top and side-view of the VIPS measurement of a live HT-29 cell are presented 15 h apart, (a) before mitosis and (b) during mitosis. Prior to mitosis (Fig. 5A), an average VIPS of points near the center of the cell are ~ 0. 56 ± 0.29°. During mitosis, cells are partially detached and cell height oscillation increases (with softness); hence a higher VIPS of ~ 0.75 ± 0.31° is observed inside the cell. Figure 5C reveals, using a t-test, statistically significant differences (*p* < 0.0001) in the pre-mitosis and during-mitosis phase shift (VIPS) values*.* This technique may be used to elucidate the homogeneity of each cell’s profile and further validates the characteristic mechanical transitioning of the cell across its growth cycle.

## Discussion

We have developed a technique to measure the time-dependent viscoelastic property changes simultaneously with cell mass accumulation over the cell cycle. This method uses a micro-resonator and measures simultaneous parameters of the resonant frequency shift, amplitude change, and mechanically driven optical path phase within a cell which is adhered to the sensor surface. This approach provides a way to characterize dynamic mechanical behavior on an individual cell basis and emphasizes the importance of time-dependent mechanical phenomena as potential biomarkers. Previous comparisons^38^ of cell mass measurements (two for adherent cells^26,37^, and one method for non-adherent cells^22^ showed the distinction between our technique and other existing ones. Our method reported in this paper is the first to provide a single cell measurement method for mass, elasticity, and viscosity on live cells across cell cycle.

The measurements have been performed using two different cell lines and we examined the trends observed from within each individual measurement; ensuring the changes are significant while drawing conclusions from each measurement. The two cell lines (HT-29 from colon cancer and MCF-7 from breast cancer) used in this study were specifically chosen as they have different characteristics in both motility and shape; which were potential contributing factors to the measurement. Even though we used two different cancer cell lines, we are able to show similar trends of the changes in viscosity and stiffness over time, indicating that our results are true at least across these specific cell lines. Although, some of our mean differences (interphase against pre-mitosis) in viscoelasticity values depict a wide variability with outliers in our measurement population. This wider spread in the data is consistent with previous similar and notable measurement schemes^1,28,29,32^,which is attributable to intrinsic discrepancies introduced during the handling of single cells.

The combination of optical measurements, measurement of resonant frequencies of the pedestal, and measurement of the vibration induced phase shift of light through the cell itself introduces many sources of noise and error. Additionally, single cell measurements lend themselves to have an expected variability across the data likely due to differences in motility, morphologies, size, and shapes. The characterization of the mechanical properties of two cancer cell lines allows us to link morphological changes with viscoelastic information across the cell cycle. Temporal viscoelasticity measurements reveal that elasticity values remain virtually unchanged from the G_1_ phase through the S phase; however, there is a significant increase in elasticity just prior to division, and then followed by a decrease in elasticity after the cell division. This is due to the well-known cortical actin stiffening effect that facilitates the division events^31^. Cell viscosity, on the other hand, shows a different trend than elasticity, with a maximum viscosity occurring during cell detachment (the actual splitting event) implying a higher sensor deflection rate due to partial adherence. After the division, cells maintain a high viscosity before being restored to the baseline viscosity values. These previously high values observed during and post-division across our entire data sets agrees with previous works on chaetopterus eggs that shows a sharp (twofold) increase in viscosity post-division^39^. This apparently occurs during the G2 ‘protein-synthesis’ phase. As a cell grows, mass is accumulated and there is an increase in the physical size, with the largest contribution to cellular dry mass being typically from proteins^40^. It is likely that the viscous component of cell behavior is influenced by the increase in proteins in the cytoplasm^41,42^. We observe a consistent and steady increase in our measurements at mitosis against interphase (*p* ≈ 0.001) as shown in Fig. 2D–E. This suggests that our device resolves the accumulation (initiation/build-up) of these proteins ~ 4–5 h prior to division, as measured by a reduced laser phase shift. These temporal viscoelastic trends agree well with an expected cortical stiffening and cellular rounding at each mitotic entrance^31^.

A current limitation of our system is that we are not able to image the changes in the contact area between the cell and the pedestal during division. This reduction in the contact area and the partial detachment of cells from their sensor-surface is correlated to the timing (~ 141 ± 19 min) of mitosis, as measured by an apparent dip in mass and peak in stiffness (and viscoelasticity). These values are obviously not accurate at the time of mitosis, but the phenomenon are real. In the future, characterization and measurement of the cell attachment on the pedestal surface can allow us to make corrections to our mass and viscoelasticity values right at mitosis.

Filamentous actin contributes to cell stiffness and the Rho kinase pathway not only directly influences actin formation^24,31^ but has also been linked with the growth and migration of cells^43,44^. Using our micro-resonator platform, we modulated single cell viscoelastic properties with drug-activated cytoskeletal changes that are also expected to change cell growth rate. On average, we observed an increase in viscoelastic properties of Rho-induced cells compared to the untreated values which agrees well with the expected development of more stress fibers^45^. The rho-treated curves exhibit a higher elasticity and viscosity overall, although, the trends over time remain similar for HT-29. Rho influences changes in growth,interestingly, when we compare an untreated MCF-7 cell with a treated MCF-7 cell, the treated cell does not divide but its growth stays static within ~ 10 h after the application of the Rho-activator, suggesting that the cell is caught in the G_0_ (resting phase) or S phase. The lack of a division event makes it challenging to compare trends in mechanical properties over the cell cycle between untreated and rho-treated MCF-7 cells.

Consistent noise levels of HT-29 (untreated: 136.4 ± 15.1 Pa, treated: 329.9 ± 17.8 Pa) and MCF-7 (untreated: 268.2 ± 23.3 Pa, treated: 525.5 ± 32.3 Pa) imply that our measurements are largely insensitive to these rho-mediated cell-sensor coupling as both rho-treated and untreated cell measurement noise levels fall within 10% of our signal measurement. Repeated measurements indicate that differences in our viscoelasticity values (interphase against pre-mitotic) are more than three times measurement uncertainty. This suggests that both elasticity and viscosity parameters can be reliably used to differentiate cell cycle phases within our representative cell types (HT-29 and MCF-7).

Our analysis relies on the underlying knowledge that rho activators have been repeatedly shown to increase cell stiffness and stress fibers. Stress fibers exhibit an interdependence with focal adhesions, whereby, an increase in stress fibers in turn increases focal adhesions. Connected focal adhesions then correspondingly act as mechanosensitive transducers in signaling the entire cell cytoskeleton network to stiffen. Specifically, they transmit forces via their receptors to and from the ECM (or sensor-surface). Hence, even if cell surface area (with sensor coupling) increases with rho-activators; they would not impact our viscoelasticity measurements. Cell instantaneous volume is always held constant irrespective of any induced cell-sensor coupling (or a higher surface area). Using our dynamic system, we have been able to extract viscoelastic mechanical information of an individual cell as it grows and progresses through the cell cycle. We further incorporated a FUCCI cell cycle reporter to optically monitor these cells and precisely correlate the stiffness and growth profile with cycle stage. The long-term measurement technique used for cell viscoelasticity and growth characterization can potentially be integrated into a multi-modal mechanical assessment of individual cells over time. Our results on live untreated and Rho-mediated cells demonstrate that we can resolve expected changes in modulus induced by forced cytoskeletal reorganization. With an enhanced throughput, and additional capabilities for imaging of cell contact area, this measurement system can make a significant contribution to understanding various cellular processes, such as cell growth, apoptosis, cell differentiation, and cell proliferation.

## Methods

### Cell-media refractive index difference and optical path length (OPL) model

To explicitly decouple the viscoelastic moduli of individual cells from a sample population, the membrane fluctuation (height oscillation) is combined with a mechanically-induced and optically-measured phase-shift of each cell using an optical path difference model. The LDV measures the time-derivative of the OPL and is used to determine the membrane oscillation of the cell^27,46,47^. Figure 1C is a schematic overview of the optical path of the laser in conjunction with difference in refractive index of each surrounding layer (air, media, cell) of its travel length. The phase shift induced during vibration is largely a function of each cell height oscillation when presented as a soft material as opposed to a rigid body. In other words, no significant phase shift is experienced when the sensor is empty, or our probed sample is not within a linear regime of compressibility (non-deformable).

The equation for the optical path length model is represented in Eq. (1) and resonant measurement parameters (amplitude ratio and mechanical phase difference) from this are shown in Eqs. (2) and (3).

1 $$\begin{aligned} {\varvec{OPL}}\left( t \right) & = \sum n_{i} d_{i} \left( t \right) = {\text{n}}_{{{\text{GM}}}} *(H_{sensor } (t) - h(t)) + {\text{n}}_{cell} *h\left( t \right) \\ & \; \approx {\text{n}}_{{{\text{GM}}}} {\text{As}}*(1 + \Delta A)*\sin (\omega {\text{t } + \text{ }}\Delta \phi ) + {\text{const}} \\ \end{aligned}$$

2 $$\Delta A = \left[ {\frac{{({\text{n}}_{cell - } {\text{n}}_{{{\text{GM}}}} ) }}{{{\text{n}}_{{{\text{GM}}}} }} *\frac{{\text{ Ac}}}{{{\text{As}}}} } \right]$$

3 $$\Delta \phi = {\arctan}\left[ {\frac{{({\text{n}}_{cell - } {\text{n}}_{{{\text{GM}}}} ) }}{{{\text{n}}_{{{\text{GM}}}} }} *\frac{{\text{ Ac}}}{{{\text{As}}}}*sin \left( \theta \right) } \right]$$

where vertical heights include $H_{sensor } \left( t \right)$ and h (t) representing the instantaneous sensor position and cell height. *n*_*cell*_ and *n*_*GM*_ represent refractive index of the cell and surrounding media respectively. *A*_*c*_ represents the amplitude of the cell height oscillation with respect to cell initial height also denoted as the cell membrane fluctuation (amplitude), *A*_*s*_ represents the amplitude of the sensor oscillation, $\theta$ represents the phase of the cell height oscillation with respect to the sensor, $\omega$ represents the oscillating resonant frequency, $\Delta \phi$ is our measured maximum phase shift of the OPL. At every time point of each long-term experiment, we compute the individual cell membrane fluctuation (cell height oscillation) at resonance, *A*_*c*_, via Eq. (2) by estimating *A*_*s*_ $\approx$ 0.29 nm (from measured velocity and oscillatory frequency) for a 35 nN excitatory input in media, *n*_*GM*_ = 1.35, and *n*_*cell*_ = 1.38 for live cells^27^.

### Cell-sensor modelling and assumptions

As in previous studies^1,24–27,47^, our sensor-cell configuration is modelled as a 2-DOF suspended mass model where the cell is represented by a Kelvin-Voigt viscoelastic solid with a mass (*m*_2_), elastic stiffness (*k*_2_), and viscous coefficient (*c*_2_) connected to the sensor. The sensor mass (*m*_1_) is also connected to the fixed substrate by a second Kelvin-Voigt spring-damper (*k*_1_, *c*_1_). The model as shown in Fig. 1D assumes an oscillatory force *F*(*t*) applied to the sensor mass.

The cell-sensor interactions used in this work depends on a few required assumptions that the cell membrane is homogeneous, smooth and the average cell-surface^24^ (membrane) oscillation is calculated at its steady state. Also, the total mass of the cell, m_*2*_ is assumed to be concentrated at the center of the cell. The displacement (and the input force) of the cell is also assumed to be applied at the center of the cell mass. Consequently, the center of the cell is in phase with the membrane of the cell; implying that $A_{c} = A_{s}$ (Supplementary information Fig. S3). We model the 2-DOF system behavior as described fully in the Supporting Information.

### Cell culture, measurement calibration and drift characterization

Commercially available HT-29 and MCF-7 (ATCC) cells were grown at 37 °C in Dulbecco’s Modified Eagles Medium supplemented with sodium pyruvate, 10% fetal bovine serum and 1% penicillin streptomycin. The cells were seeded onto the sensor area at a density of ~ 300 cell/mm^2^ within a 6 mm diameter PDMS culture chamber. Cells were cultured on resonant sensors functionalized with collagen. A separate cell sample cells was treated with 2 μg/mL of Rho Activator II (CN03, Cytoskeleton, Inc.) and incubated for 4 h before and during our mechanical measurements. We, also collected instantaneous amplitude ratio^24^^,^ phase shift^27^^,^ and frequency shift^26^ measurements, calibrated the sensor mass, and characterize long-term drifts^1,24–27^ (also see Supporting Information) to estimate the viscoelasticity of each live cell and fully understand the underlying mechanics of each cell as they progress across their various growth stages from interphase through mitosis.

### FUCCI cell cycle marker

In order to capture the transitions through the cell cycle, we used commercially available (Thermo Fisher Scientific) fluorescence ubiquitination cell cycle indicator (FUCCI), a genetically encoded, dual-colored (red and green) fluorescent probe that allows us to visualize each cell cycle progression within our cell population. During the G1 S transition of a single cell, a color transition occurs, from red-through-yellow-to-green, indicating the progression through its cell cycle and division as shown in Fig. 1B (right) for an MCF-7 cell. A 20 μL dilution mix of the FUCCI BacMam reagents (geminin-GFP and Cdt1-RFP) was added to 50,000 adherent cells targeting 40 particles per cell. The reagent is a baculovirus, an insect virus that does not replicate in mammalian cells. After gentle rotation, we incubated the cells overnight (≥ 16 h) at 37 °C and then fluorescence imaging was carried out to measure the cell cycle transition.

### Imaging of cultured cells (Olympus BX 51)

Cells were grown on our sensors and stably transduced with the FUCCI BacMam reagents. They were then subjected to long-term, time-lapse imaging using a computer-assisted fluorescence microscope (Olympus, BX 51, cellsens software) equipped with an objective lens (20X Olympus Plan Achromat Objective, 0.4 NA, 1.2 mm WD), a halogen lamp (excitation source), and a CCD camera. For fluorescence imaging, the halogen lamp was used with two filter cubes FITC and TRITC. The FITC filter with an excitation maximum wavelength of 490 nm and emission maximum wavelength of 525 nm was used to observe the fluorescence of Premo geminin-GFP. The TRITC filter with an excitation maximum wavelength of 557 nm and emission maximum wavelength of 576 nm was used to observe the fluorescence of Premo Cdt1-RFP.

Figure 3A describes the transition between the Olympus BX51 fluorescence microscope and LDV stage as each cell progresses across its cycle. The cells were collected intermittently from the LDV stage to the microscope for imaging at different time windows of the growth stage. For instance, since it takes 6–12 h to transition from stage G_0_/G_1_ to the S growth phase. The MCF-7 cells were quickly imaged on our upright fluorescence microscope within the smallest detectable measurement time frames of ~ 5–10 min before resuming the cell mass/stiffness measurements. The same procedure was carried out through phases G_0_ /G_1_–S to G_2_–M transitions.

### Estimation of instantaneous cell viscoelastic coefficients, (k_2_, c_2_) and moduli (E, μ)

The mechanical phase difference ($\theta$) is related to cell viscoelastic properties that we extract by considering the full 2-DOF suspended mass system. To estimate the viscoelastic coefficients (*k*_2_, *c*_2_), our micro-resonator is modeled as a 2-DOF Kevin–Voigt model as described in Eq. (4) in simplified matrix form.

4 $$\left[ {\begin{array}{*{20}c} {(k_{1} + k_{2} - m_{1} \omega^{2} ) + (c_{1} + c_{2} )\omega j} & { - k_{2} - c_{2} \omega j} \\ { - k_{2} - c_{2} \omega j} & {\left( {k_{2} - m_{2} \omega^{2} } \right) + c_{2} \omega j} \\ \end{array} } \right]\left\{ {\begin{array}{*{20}c} {{\varvec{A}}_{{\varvec{s}}} } \\ {{\varvec{A}}_{{\varvec{c}}} } \\ \end{array} } \right\}e^{j\omega t} = \left\{ {\begin{array}{*{20}c} F \\ 0 \\ \end{array} } \right\}{ }e^{j\omega t} ;$$

The physical quantities of interest are found by taking the imaginary components, e.g. $F\left( t \right) = F\sin \omega t = {\text{I}}_{{\text{m}}} \left\{ {Fe^{j\omega t} } \right\}$; where ${\varvec{A}}_{{\varvec{s}}} = A_{s}^{R} + jA_{s}^{I}$ and ${\varvec{A}}_{{\varvec{c}}} = A_{s}^{R} + jA_{c}^{I}$ are the vector amplitudes of the sensor and cell states $x_{sensor}$ and $x_{cell}$ respectively, and $Fe^{j\omega t}$ is the input force. Equation (4) is further decomposed as the instantaneous sensor and cell responses in Eq. (5).

5a $$x_{sensor} \left( t \right) = \left| {{\varvec{A}}_{{\varvec{s}}} } \right| \sin \left( {\omega t + \theta_{s} } \right)$$

5b $$x_{cell} \left( t \right) = \left| {{\varvec{A}}_{{\varvec{c}}} } \right| \sin \left( {\omega t + \theta_{c} } \right)$$

where $$\theta_{s} = \tan^{ - 1} \left\{ {{\raise0.7ex\hbox{${(A_{s}^{I} )}$} \!\mathord{\left/ {\vphantom {{(A_{s}^{I} )} {(A_{s}^{R} )}}}\right.\kern-\nulldelimiterspace} \!\lower0.7ex\hbox{${(A_{s}^{R} )}$}}} \right\}$$ and $$\theta_{c} = \tan^{ - 1} \left\{ {{\raise0.7ex\hbox{${(A_{c}^{I} )}$} \!\mathord{\left/ {\vphantom {{(A_{c}^{I} )} {(A_{c}^{R} )}}}\right.\kern-\nulldelimiterspace} \!\lower0.7ex\hbox{${(A_{c}^{R} )}$}}} \right\}$$. Here, $$\theta_{s}$$ and $$\theta_{c}$$ denote the phase differences between the sensor and cell height oscillation with respect to the excitatory force, $$F\left( t \right) = F\sin \omega t$$. Our modeled cell-sensor phase difference,

6 $$\theta = \theta_{c} - \theta_{s}$$

and modeled cell-sensor amplitude ratio becomes:

7 $$\Delta {\text{A}} = \frac{{({\text{n}}_{cell - } {\text{n}}_{{{\text{GM}}}} ) }}{{{\text{n}}_{{{\text{GM}}}} }} \frac{{\left| {{\varvec{A}}_{{\varvec{c}}} } \right|}}{{\left| {{\varvec{A}}_{{\varvec{s}}} } \right| }}$$

Observed amplitude ratio ($\Delta A$) and mechanical phase shift ($\theta)$ inferred from Eq. (2) are substituted to simultaneously solve Eqs. (6) and (7) for *k*_2_ and *c*_2_.

The viscoelastic coefficients ($$k_{2} , c_{2} )$$can be related to the apparent inherent viscoelastic moduli (*E, μ*) by $$k_{2} = \frac{EA}{H}$$ and $$c_{2} \frac{{{\upmu }A}}{H}$$. Ratio $$\frac{A}{H}$$ denotes the area-to-height information of each cell where the average cell area is ~ 250 µm^2^ with an estimated cell height of ~ 8 µm and fitted accordingly in previous work^19^.

### Interplay between the corrected mass and stiffness values

Prior studies^1,24–27^, demonstrate that our 2-DOF model represented by Eq. (4) shows that the measured apparent mass in Fig. 3B–C, is a function of the cell viscoelastic moduli. Thus, all measured mass values in the remainder of this work were corrected by a coupling factor of (1/0.6) to account for the finite stiffness of the cell and in turn, factor in any possible cell-sensor decoupling at every time instant. This only changes with slight variations in the cell’s viscoelasticity (stiffness) and morphology. Particularly, Fig. 3C–D highlights how the rho-mediated cells exhibit high apparent (uncorrected) elasticities comparable to its corrected (untreated) counterparts in Fig. 2D–E. This suggests an increased cell-sensor coupling ~ 30 min after the rho-drug was added.

### Vibration induced phase shift (VIPS)

The MEMS resonant platform sensor is designed to vibrate vertically for uniform mass sensitivity^26^. The vibration produces an oscillation in cell height that is inversely proportional to cell stiffness. Similar to previous work^27^^,^ we stimulated our platform at a fixed frequency with an amplitude of ~ 290 pm and the VIPS is measured by a LDV at three different locations (1–3) outside the cell and one location (4) inside the cell, as shown in Supplementary information Fig. S8. Beyond single point measurements we scanned over the entire cell to obtain the 2-dimensional measurement and visualization of VIPS (Fig. 5). It was observed that the phase shift of soft cell gradients is higher than that of stiffer cell gradient. This is because the phase shift is directly proportional to cell height oscillation as observed in Eqs. (3) and (7). Details of this analysis can be found in other studies^27^_._ Prior to mitosis, we observed in Fig. 5A an increase in stiffness and a corresponding low average phase shift (VIPS) near the center of the cell are of ~ 0. 56 ± 0.21°. During mitosis Fig. 5B, while cells are partially detached, the cell increases in height oscillation (and softness); hence a higher average VIPS of 0.75 ± 0.31°*.* A statistically significant (*p* < 0.0001) difference in pre-mitosis (before-mitosis) and during mitosis is presented in Fig. 5C. These VIPS measurement profile between ~ 180 scanned points are linearly interpolated as shown and scaled in the color bars. The grid-based technique helps in elucidating the morphological changes and heterogeneity of each cell’s profile and further validates our observed characteristic transitioning of a cell across its growth cycle.

## Supplementary information

Supplementary information
