# Supplementary material for: Simultaneous time-varying viscosity, elasticity, and mass measurements of single adherent cancer cells across cell cycle
Source: Sci Rep. 2020 Jul 30;10:12803. doi: 10.1038/s41598-020-69638-z (PMC7393350; doi:10.1038/s41598-020-69638-z)
Supplement: Supplementary file 1 — Supplementary information [file 41598_2020_69638_MOESM1_ESM.pdf]

Simultaneous Time-Varying Viscosity, Elasticity, and Mass Measurements of Single Adherent Cancer Cells across Cell Cycle

Olaoluwa O. Adeniba[1,2,†], Elise A. Corbin[3,4,†], Anurup Ganguli[2,5], Yongdeok Kim[2,6], and Rashid Bashir[1,2,5,7*]

[1]*Department of Mechanical Science and Engineering, University of Illinois Urbana-Champaign, Urbana, IL 61801*

[2]*Micro and Nanotechnology Laboratory, University of Illinois Urbana-Champaign, Urbana, IL 61801*

[3]*Biomedical Engineering Department, University of Delaware, Newark, DE 19716*

[4]*Materials Science and Engineering Department, University of Delaware, Newark, DE 19716*

[5]*Department of Bioengineering, University of Illinois Urbana-Champaign, Urbana, IL 61801*

[6]*Department of Materials Science and Engineering, University of Illinois Urbana-Champaign, Urbana, IL 61801*

[7]*Carle Illinois College of Medicine, University of Illinois Urbana-Champaign, Urbana, IL 61801*

† equal contribution

* rbashir@illinois.edu

Supplementary Note 1: **Characterizing drifts in Amplitude Ratios and Frequency Shifts**

In this work, amplitude and frequency measurements of loaded sensors are taken every ~3 minutes while cells grow; however, the amplitude ratio of empty sensors needs to be monitored as well. It is observed that over time, the sensors exhibit a negative drift in amplitude and resonant frequency (100 – 150 Hz). We compensate for this drift by simultaneously measuring empty neighboring sensors throughout the observation period of ~24hrs. Correcting for drift effects in the amplitude and frequency measurement involves estimating these parameters as measured by equivalent empty sensors and subtracting from the measurements taken from loaded sensors.

Supplementary Note 2: **Nanoscale Membrane Fluctuation Estimation**

At every hourly time point of each long-term experiment, we compute the individual cell membrane fluctuation at resonance, A_c, for one cell per sensor. This is calculated via equation (2) by estimating $A_s = 0.29$ nm for a 35 nN excitatory input in media, $n_{GM} = 1.35$, and $n_{cell} = 1.38$ for live cells. To better understand how the changes in cell membrane fluctuations occur, we modeled our sensor-cell system as a 2-DOF suspended mass model (**fig. S7a**) where the cell mass (m_2) is considered a Kelvin-Voigt viscoelastic solid with elastic stiffness (k_2) and viscous coefficient (c_2) connected to the sensor. The sensor mass (m_1) is also connected to the fixed substrate by a second Kelvin-Voigt spring-damper (k_1, c_1). The model assumes an oscillatory force $F(t)$ applied to the sensor mass.

Fig. S7b shows the instantaneous cell height oscillation with respect to the sensor, $h(t) = h_0 + A_C \sin(\omega t + \theta_c)$; A_C denotes the amplitude of the cell height with respect to the static height, h_0 (membrane fluctuation), θ_c denotes the phase difference between cell height oscillation

with respect to the applied force and A_s represents the amplitude of the sensor oscillation at resonance frequency, ω.

Supplementary Note 3: **Estimation of Viscoelastic Coefficients, (k, c) for each time step**

As already solved and established in our previous study[4], our cell-sensor system is simply modelled as the equations of motion of a 2DOF Kevin-Voigt model written in matrix form is:

$$\begin{bmatrix} m_1 & 0 \\ 0 & m_2 \end{bmatrix} \begin{Bmatrix} \ddot{x}_s \\ \ddot{x}_c \end{Bmatrix} + \begin{bmatrix} c_1 + c_2 & -c_2 \\ -c_2 & c_2 \end{bmatrix} \begin{Bmatrix} \dot{x}_s \\ \dot{x}_c \end{Bmatrix} + \begin{bmatrix} k_1 + k_2 & -k_2 \\ -k_2 & k_2 \end{bmatrix} \begin{Bmatrix} x_s \\ x_c \end{Bmatrix} = \begin{Bmatrix} F \\ 0 \end{Bmatrix} \boldsymbol{e^{j\omega t}} \qquad (4)$$

where the spring constant k_1 and the resonant frequency ω are measured in dry air for each sensor.

The input forcing $F(t) = F \sin \omega t$ is represented as $Fe^{j\omega t}$; in our analysis below with complex notation, the resulting imaginary components represent the physical quantities of interest, e.g. $F(t) = F \sin \omega t = \mathrm{I_m}\{Fe^{j\omega t}\}$. Let the steady state solution assume the form:

$$\boldsymbol{x(t)} = \mathbf{A}e^{j\omega t} = \begin{bmatrix} \boldsymbol{A_s} \\ \boldsymbol{A_c} \end{bmatrix} e^{j\omega t} \qquad (5)$$

where the sensor and cell amplitude vectors are, $\boldsymbol{A_s} = A_s^R + jA_s^I$ and $\boldsymbol{A_c} = A_s^R + jA_c^I$. Here, A^R and A^I denote real and imaginary components of amplitude, A. Substituting (5) into (4) yields:

$$\begin{bmatrix} (k_1 + k_2 - m_1\omega^2) + (c_1 + c_2)\omega j & -k_2 - c_2\omega j \\ -k_2 - c_2\omega j & (k_2 - m_2\omega^2) + c_2\omega j \end{bmatrix} \begin{Bmatrix} A_s \\ A_c \end{Bmatrix} e^{j\omega t} = \begin{Bmatrix} F \\ 0 \end{Bmatrix} e^{j\omega t} \qquad (6)$$

Dividing (6) by the nonzero scalar $e^{j\omega t}$ yields a complex matrix equation in the amplitude, $\boldsymbol{A_s}$ and $\boldsymbol{A_c}$. We calculate the matrix inverse using:

$$A^{-1} = \frac{Cofactor\ (A)}{\det(A)} \qquad (7)$$

where the cofactor of matrix, $A = \begin{bmatrix} a & b \\ c & d \end{bmatrix}$ is $\begin{bmatrix} d & -b \\ -c & a \end{bmatrix}$ and $det\ (A) = ad - bc$

Calculating the matrix inverse of using equation 7 and multiplying by 6 yields:

$$\begin{bmatrix} \mathbf{A_s} \\ \mathbf{A_c} \end{bmatrix} = \frac{\begin{bmatrix} (k_1 + k_2 - m_1\omega^2) + (c_1 + c_2)\omega j & -k_2 - c_2\omega j \\ -k_2 - c_2\omega j & (k_2 - m_2\omega^2) + c_2\omega j \end{bmatrix} \begin{bmatrix} F_0 \\ 0 \end{bmatrix}}{\det(\boldsymbol{K} - \omega^2\boldsymbol{M} + \omega j\boldsymbol{C})} \tag{8}$$

Here, the determinant in the denominator is given by:

$$\det(\boldsymbol{K} - \omega^2\boldsymbol{M} + \omega j\boldsymbol{C}) = m_1 m_2\,\omega^4 - (c_1 c_2 + m_2\,(k_1 + k_2) + k_2\,m_1)\omega^2 + k_1 k_2 + + [(k_1 c_2 + c_1 k_2)\omega - (c_2\,(m_1 + m_2) + c_1 m_2)\,\omega^3] j \tag{9}$$

And the system coefficient matrices, $\boldsymbol{M}, \boldsymbol{C},$ and $\boldsymbol{K}$ are given by:

$$\boldsymbol{M} = \begin{bmatrix} m_1 & 0 \\ 0 & m_2 \end{bmatrix}, \quad \boldsymbol{C} = \begin{bmatrix} c_1 + c_2 & -c_2 \\ -c_2 & c_2 \end{bmatrix} \text{ and } \boldsymbol{K} = \begin{bmatrix} k_1 + k_2 & -k_2 \\ -k_2 & k_2 \end{bmatrix} \tag{10}$$

Simplifying the matrix vector product yields:

$$\boldsymbol{A_s} = \frac{[(k_2 - m_2\omega^2) + c_2\,\omega j]F}{\det(\boldsymbol{K} - \omega^2\boldsymbol{M} + \omega j\boldsymbol{C})} \tag{11}$$

$$\boldsymbol{A_c} = \frac{[(k_2 + c_2\,\omega j)]F}{\det(\boldsymbol{K} - \omega^2\boldsymbol{M} + \omega j\boldsymbol{C})} \tag{12}$$

We establish that $x_{sensor}(t) = |\boldsymbol{A_s}|\,\sin(\omega t + \theta_s)$ and $x_{cell}(t) = |\boldsymbol{A_c}|\,\sin(\omega t + \theta_c)$ while $\theta_s = \tan^{-1}\left\{ (A_s^I) / (A_s^R) \right\}$, $\theta_c = \tan^{-1}\left\{ (A_c^I) / (A_c^R) \right\}$; where, θ_s and θ_c denote the phase differences between the sensor and cell height oscillation with respect to the excitatory force, $F(t) = F_0 \sin \omega t$. Thus, our modeled cell-sensor phase difference,

$$\theta = \theta_c - \theta_s = f_1(m_1, m_2, c_1, c_2, k_1, k_2, \omega) \tag{13}$$

and our modeled amplitude ratios become:

$$\varDelta \mathrm{A} = \frac{(\mathrm{n}_{cell} - \mathrm{n}_{\mathrm{GM}})}{\mathrm{n}_{\mathrm{GM}}}\ \frac{|\boldsymbol{A_c}|}{|\boldsymbol{A_s}|} = f_2(m_1, m_2, c_1, c_2, k_1, k_2, \omega) \tag{14}$$

For all the long-term cell-to-cell data-points obtained, we compare experimental data ($\varDelta$A and $\varDelta\phi$) obtained in obtained in equations (2) and (3) into (13) and (14) to simultaneously solve for our cell's viscoelastic coefficients, c_2 and k_2.

The elasticity, E and viscosity, μ are then obtained by using the following conversions:

$$k_2 = \frac{EA}{H} \tag{15}$$

$$c_2 = \frac{\mu A}{H} \tag{16}$$

Cross-sectional area, A and average cell height, H are extrapolated from our data and images[1]. The error bars on the elasticity and viscosity values in Figure 2(D) and (E) are obtained through the effects on the variables uncertainty on f_1, $and\ f_2$. Thus, viscoelastic moduli errors can be calculated as:

$$\delta E = f_3(\delta\omega, \delta A, \delta H, \delta k_2) \tag{17}$$

$$\delta\mu = f_4(\delta\omega, \delta A, \delta H, \delta c_2) \tag{18}$$

Addition, multiplication and division methods on propagation of uncertainties were applied on (13) and (14) above to obtain the error bars generated in previous work. Frequency shift uncertainties, $\delta\omega$ inherently captures the uncertainties in cell and sensor masses (m_2, m_1). Overall, uncertainties can be obtained based on the unknown system parameters and our used model.

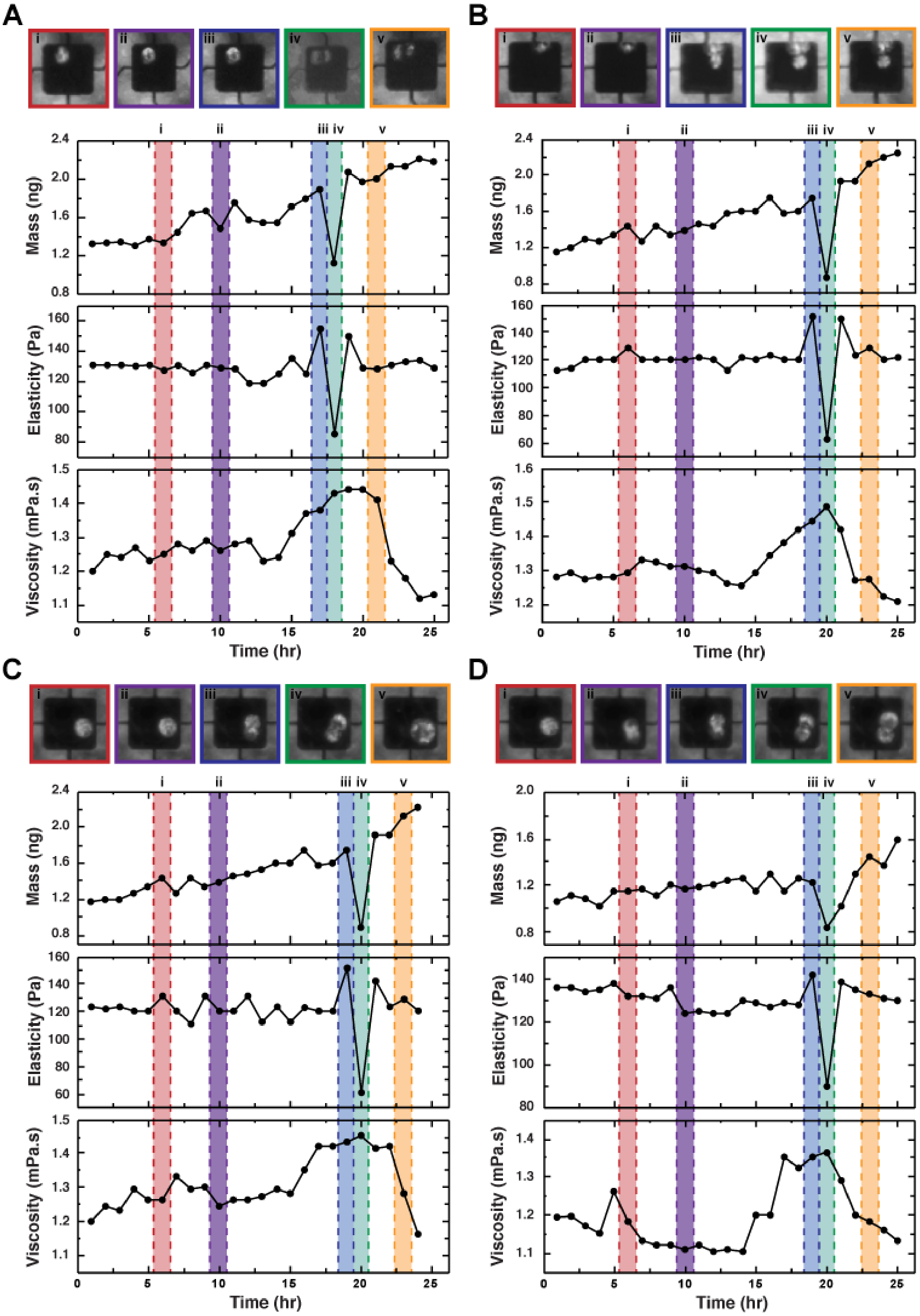
A
B
C
D
i
ii
iii
iv
v
Mass (ng)
Elasticity (Pa)
Viscosity (mPa.s)
Time (hr)

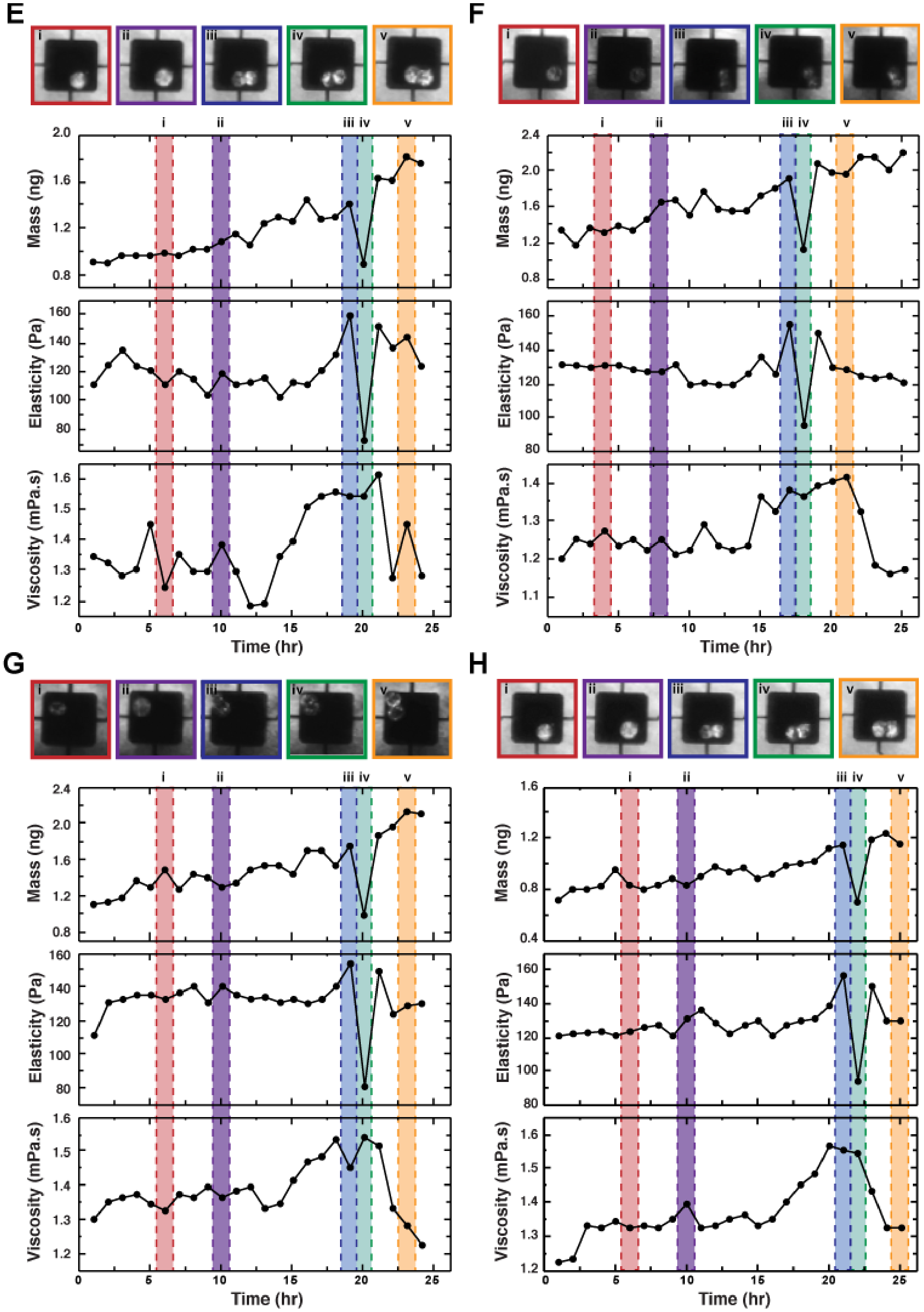

E
F
G
H
i
ii
iii
iv
v
Mass (ng)
Elasticity (Pa)
Viscosity (mPa.s)
Time (hr)

Figure S1. (A-H) HT-29 Cell mass, elasticity and viscosity versus time. (i) For single cell growth analysis of HT-29 cells, the mass and stiffness data was analyzed prior to- (i, ii, and iii – orange, brown, blue, respectively) and after a mitotic event (iv and v – green and yellow, respectively). Division is shown with the individual cells and daughter cells.

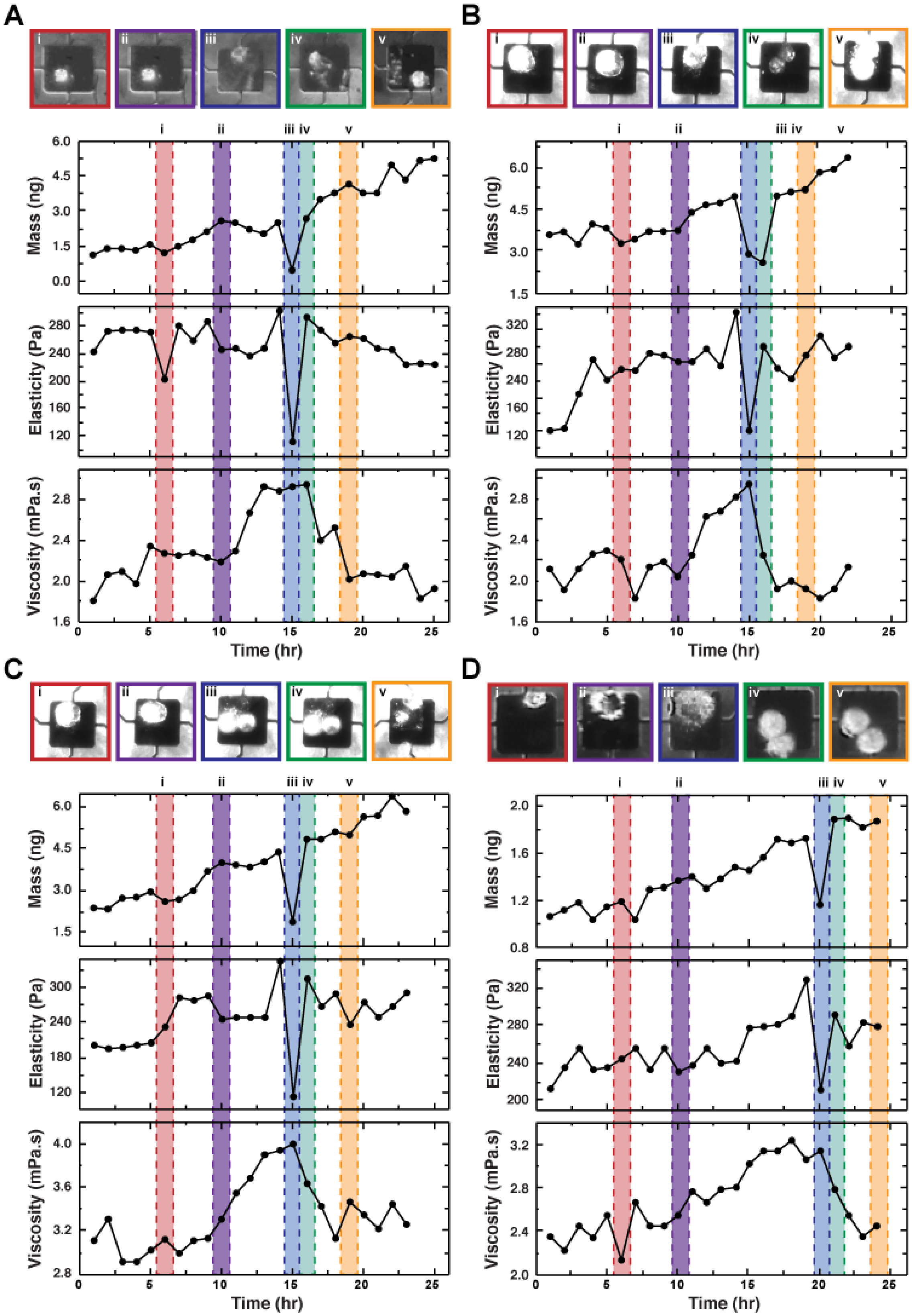
A
B
C
D
i
ii
iii
iv
v
Mass (ng)
Elasticity (Pa)
Viscosity (mPa.s)
Time (hr)

Figure S2. (A-D) MCF-7 cell mass, elasticity and viscosity versus time. (i) For single cell growth analysis of MCF-7 cells, the mass and stiffness data was analyzed prior to- (i, ii, and iii – orange, brown, blue, respectively) and after a mitotic event (iv and v – green and yellow, respectively). Division is shown with the individual cells and daughter cells.

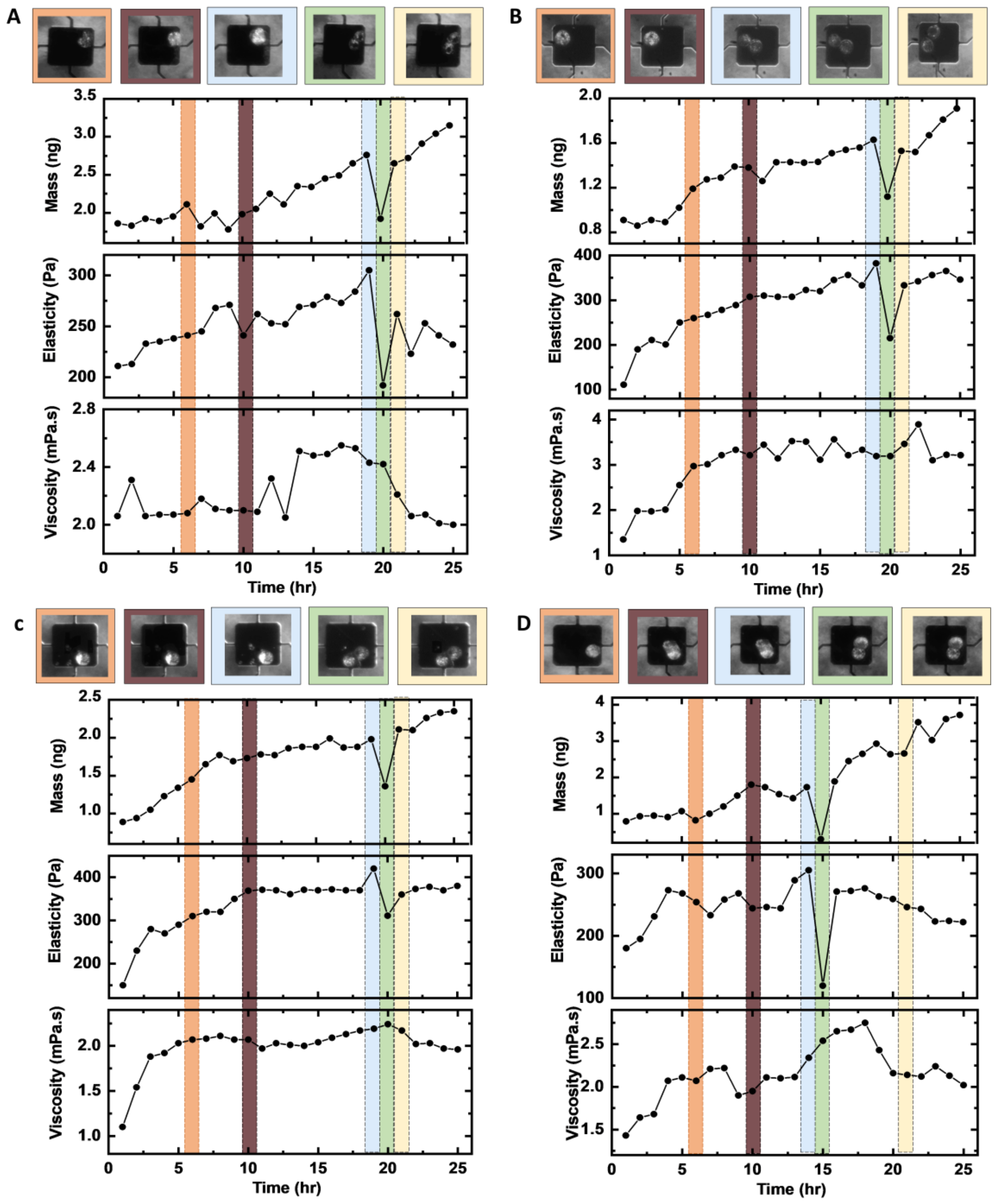

Figure S3. (A-D) HT-29 Rho-treated cell mass, elasticity and viscosity versus time. (i) For single cell growth analysis of MCF-7 cells, the mass and stiffness data was analyzed prior to- (i, ii, and iii – orange, brown, blue, respectively) and after a mitotic event (iv and v – green and yellow, respectively). Division is shown with the individual cells and daughter cells.

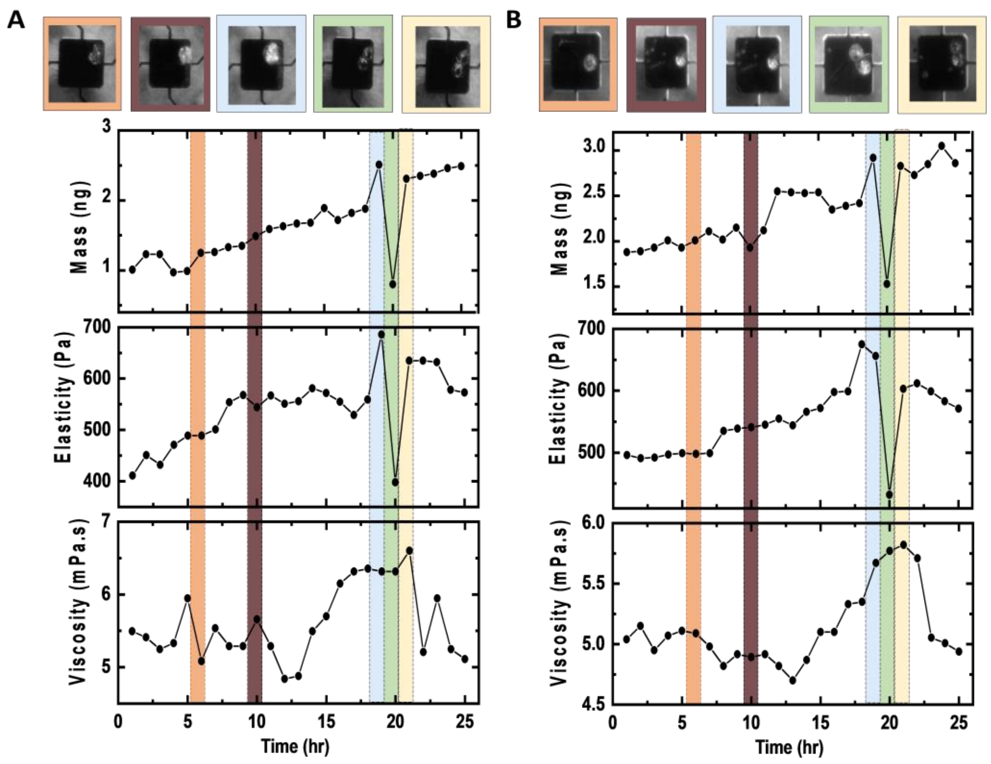

Figure S4. (A-B) MCF-7 Rho-treated cell mass, elasticity and viscosity versus time. (i) For single cell growth analysis of MCF-7 cells, the mass and stiffness data was analyzed prior to- (i, ii, and iii – orange, brown, blue, respectively) and after a mitotic event (iv and v – green and yellow, respectively). Division is shown with the individual cells and daughter cells.

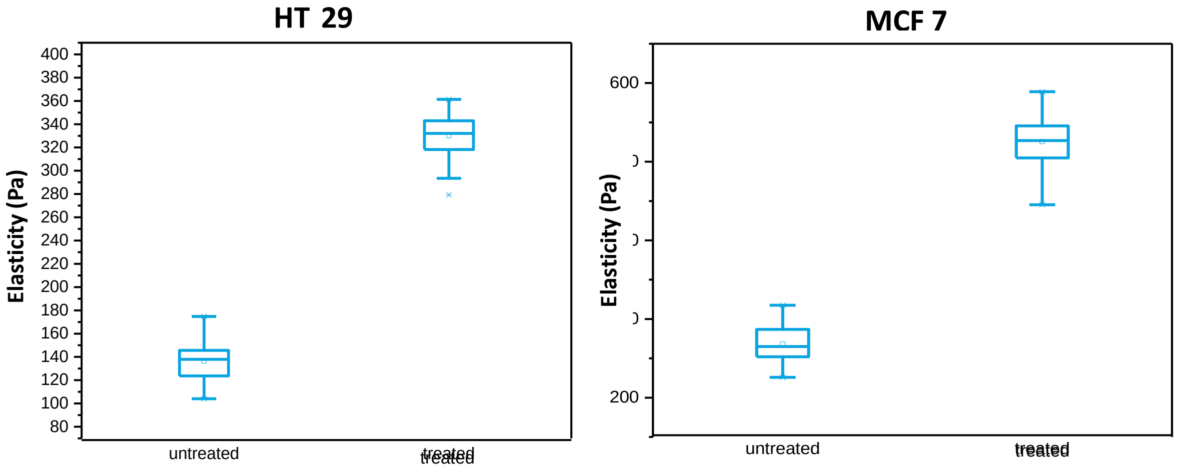

Figure S5. Comparison of measurement sensitivity of rho-treated and untreated cell elasticity with 80 repeated measurements. Data shows noise level of both rho-treated and untreated is consistent – within one order of magnitude. This elucidating the our measurements are minimally sensitive to rho-induced cell-sensor coupling. Data presented as a mean ± standard error of the mean.

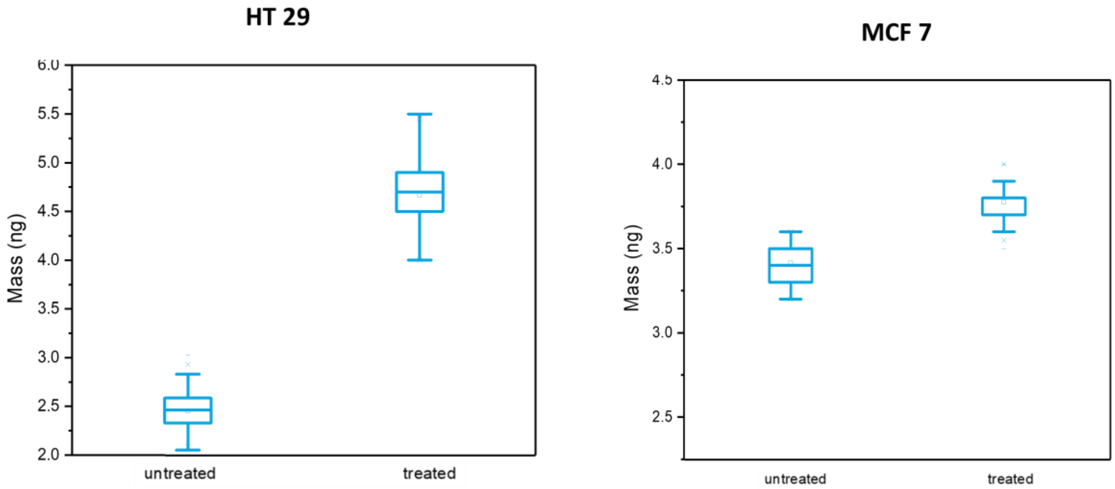

Figure S6. Comparison of measurement sensitivity of rho-treated and untreated cell mass with 100 repeated measurements. Data shows noise level of both rho-treated and untreated is consistent – within 10%. This elucidating the our mass measurements are minimally sensitive to rho-induced

cell-sensor coupling. Data presented as a mean ± standard error of the mean.

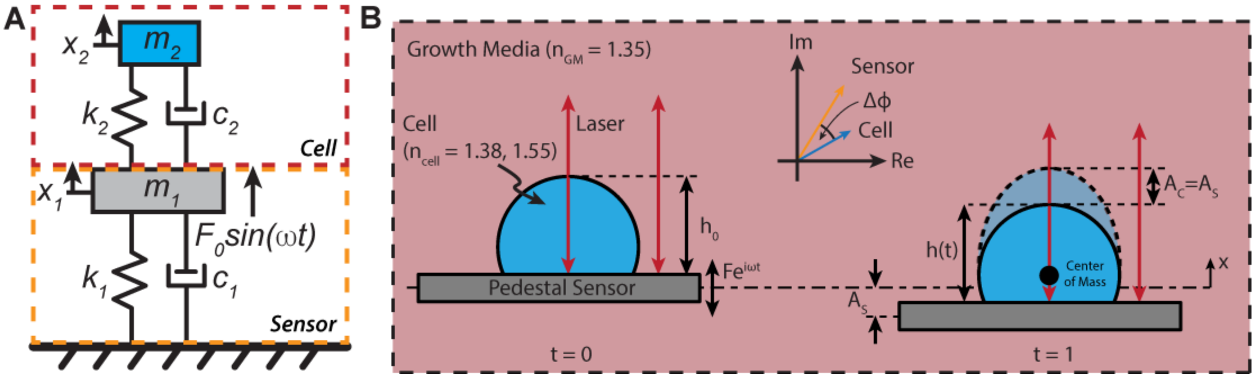

Figure S7. (A) Model of sensor-cell system as a 2-DOF suspended mass model where the cell mass (m_2) is considered a Kelvin-Voigt viscoelastic solid with elastic stiffness (k_2) and viscous coefficient (c_2) connected to the sensor, and the sensor mass (m_1) is connected to the fixed substrate by a second Kelvin-Voigt spring-damper (k_1, c_1). The model assumes an oscillatory force F(t) applied to the sensor mass. (B) Schematic depicting two-time steps of the applied stimuli ($Fe^{j\omega t}$). *Left:* Pedestal sensor supporting cell with static height, h_0 and a refractive index, n_{cell}. *Right:* Showing the instantaneous cell height oscillation with respect to the sensor, $h(t) = h_0 + A_C \, sin(\omega t + \theta_c)$; A_C denotes the amplitude of the cell height with respect to the static height, h_0 (membrane fluctuation), θ_c denotes the phase difference between cell height oscillation with respect to the applied force and A_s represents the amplitude of the sensor oscillation at resonance frequency, ω. Shift ($\Delta\phi$) indicates the observed optical phase shift between two light paths, one through the cell and the other directly on the sensor (red lines with arrows)[2].

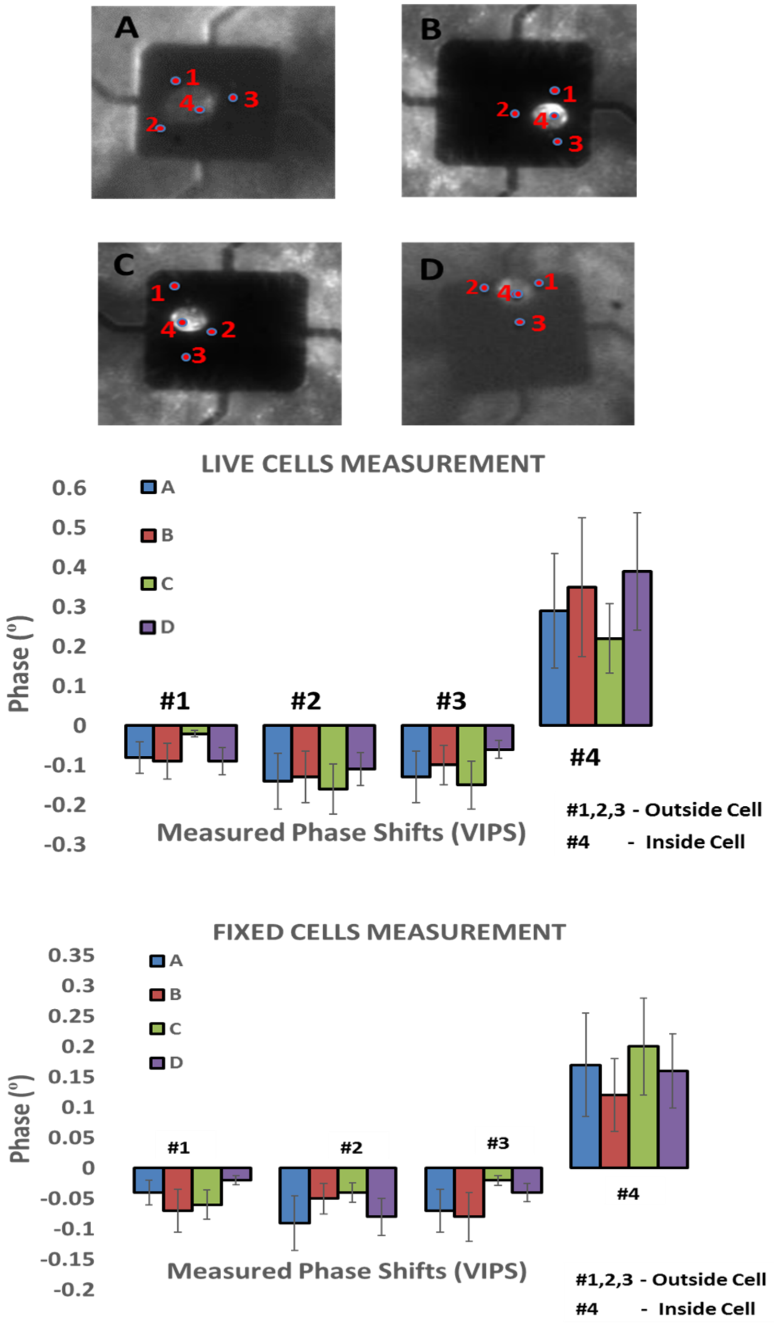

Figure S8. Typical raw data of observed phase shift by incident laser on outside cell: #1, #2 and #3 versus inside of cells: #4 for live cells; A, B, C, D and their corresponding fixed counterparts measured at 1Vrms. Average phase shift shows ~0.39 ± 0.11° for live cells and 0.19 ± 0.09° for fixed cell.[2,3] Data presented as a mean ± standard deviation.

Reference

1. Park, K., Millet, L. J., Kim, N., Li, H., Jin, X., Popescu, G., Aluru, N. R., Hsia, K. J. & Bashir, R. Measurement of Adherent Cell Mass and Growth. *Proc. Natl. Acad. Sci.* **107**, 20691–20696 (2010).
2. Park, K., Mehrnezhad, A., Corbin, E. A. & Bashir, R. Optomechanical Measurement of the Stiffness of Single Adherent Cells. *Lab Chip* (2015). doi:10.1039/C5LC00444F
3. Mehrnezhad, A. & Park, K. Multifrequency Optomechanical Stiffness Measurement of Single Adherent Cells on a Solid Substrate with High Throughput. *Anal. Chem.* **89**, 10841–10849 (2017).
4. Adeniba, O. O., Corbin, E. A., Ewoldt, R. H. & Bashir, R. Optomechanical Microrheology of Single Adherent Cancer Cells. *APL Bioeng.* **2**, 016108 (2018).
